# Knowledge, health-seeking behavior, and predictors of treatment adherence among tuberculosis patients in the North-West Region of Cameroon

**DOI:** 10.11604/pamj.2026.53.165.48556

**Published:** 2026-04-20

**Authors:** Ndukong Nsanwe Ndi, Moses Samje, Mbuwir Bongfen Charlotte, Mary Bi Suh Atanga

**Affiliations:** 1 Department of Public Health, University of Bamenda, Bamenda, Cameroon,; 2 Department of Biomedical Sciences, University of Bamenda, Bamenda, Cameroon,; 3 Department of Nursing and Midwifery, University of Bamenda, Bamenda, Cameroon

**Keywords:** Tuberculosis, treatment adherence, health-seeking behaviour, Cameroon

## Abstract

**Introduction:**

tuberculosis (TB) remains a major public health challenge globally, with sub-Saharan Africa bearing a disproportionate burden, and control efforts in the North-West Region of Cameroon have been severely disrupted by prolonged socio-political instability. The objective of this study aimed to assess tuberculosis knowledge, describe health-seeking behavior, and identify predictors of treatment adherence among tuberculosis patients receiving treatment in the North-West Region of Cameroon.

**Methods:**

a facility-based cross-sectional study was conducted among 274 adult TB patients receiving treatment in eight high-volume diagnostic and treatment centres in the North-West Region between March 2024 and March 2025. Data was collected using a semi-structured questionnaire covering sociodemographic characteristics, tuberculosis knowledge, health-seeking behaviour, treatment adherence, and patient experiences during treatment. Descriptive statistics were used to summarize the data. Associations between variables and treatment adherence were examined using chi-square tests, while multivariable logistic regression was used to identify independent predictors of adherence.

**Results:**

overall, 48.4% of respondents demonstrated adequate knowledge of tuberculosis. Male gender (p<0.001) and higher education level (p=0.003) were significantly associated with better TB knowledge. Prior to seeking formal care, 88% of participants reported self-medication. Treatment adherence was reported by 70.1% of participants. Secondary education (AOR = 2.03; 95% CI: 1.08-3.84; p=0.028) and employment status (AOR = 1.78; 95% CI: 1.04-3.03; p=0.035) were identified as independent predictors of adherence. Drug shortages, difficulties accessing treatment centres, and financial constraints were commonly reported barriers to adherence.

**Conclusion:**

tuberculosis knowledge among patients in the North-West Region of Cameroon remains suboptimal, and treatment adherence is influenced by socioeconomic and health system barriers. Strengthening patient education and ensuring uninterrupted access to treatment are critical for improving TB control in conflict-affected settings.

## Introduction

Tuberculosis (TB) remains one of the leading infectious causes of morbidity and mortality worldwide, particularly in resource-limited settings. In 2021, the World Health Organization estimated that approximately 10.6 million people developed TB globally, and about 1.6 million deaths were attributed to the disease. The African Region continues to carry a disproportionate share of the burden, largely driven by poverty, HIV co-infection, and weak health system capacity [1]. Despite global progress in TB control, drug-resistant tuberculosis and TB-HIV co-infection continue to threaten recent gains in many high-burden countries [2].

Sub-Saharan Africa remains particularly affected, with many countries facing the combined challenges of high TB incidence and limited health system resources. Cameroon is among the countries with a high burden of TB HIV co-infection, which complicates disease management and treatment outcomes [3]. To address this burden, Cameroon´s National Tuberculosis Control Programme adopted the World Health Organization-recommended ´Directly Observed Treatment Short Course´ strategy in the early 2000s to strengthen case detection and ensure treatment completion [4]. However, achieving optimal TB control requires more than clinical interventions alone. Patient-related factors such as knowledge of tuberculosis, patterns of health-seeking behavior, and adherence to treatment play an important role in determining treatment outcomes [5-7].

Evidence from several settings shows that limited knowledge of TB, delays in seeking care and treatment interruptions can negatively affect TB control efforts. Studies conducted in Nigeria, Bangladesh, and Nepal have demonstrated that patient knowledge and health-seeking behaviors significantly influence adherence to tuberculosis treatment [8-10]. The situation is further complicated in the North-West Region of Cameroon, where ongoing socio-political instability has disrupted health service delivery and limited access to care. These disruptions can lead to diagnostic delays, treatment interruptions, and reduced patient engagement with health services. TB control in the region relies on a network of diagnostic and treatment centres that provide diagnosis and treatment services across multiple health districts. While these facilities play an important role in delivering TB care, there is limited evidence on how patient knowledge, health-seeking behaviour, and treatment adherence interact in this fragile setting.

Understanding these factors is important for designing patient-centred strategies that improve treatment outcomes and strengthen tuberculosis control in conflict-affected areas. However, evidence on these behavioural and social determinants among TB patients in the North-West Region of Cameroon remains limited.

**Objective of the study:** the objective of this study was to examine tuberculosis knowledge, health-seeking behavior, and treatment adherence among TB patients receiving treatment in the North-West Region of Cameroon.

**Specific objectives:** 1) to determine the level of tuberculosis knowledge among TB patients receiving treatment in the North-West Region of Cameroon; 2) to describe health seeking behavior among TB patients prior to diagnosis; 3) to estimate the proportion of TB patients adhering to anti-tuberculosis treatment; 4) to identify socio-demographic factors associated with treatment adherence; 5) to describe patient-reported experiences and barriers encountered during tuberculosis diagnosis and treatment.

## Methods

**Figure 1:**
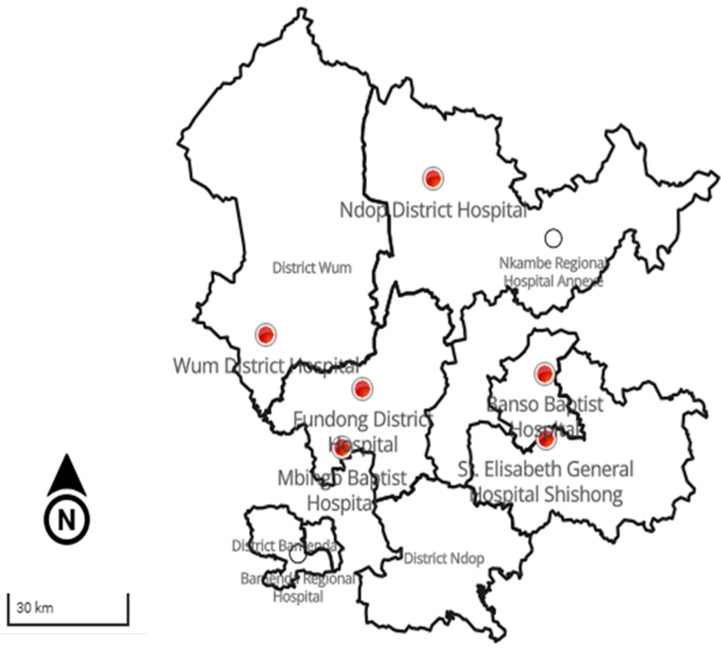
distribution of patients across selected diagnostic and treatment centers in North-West Cameroon

**Study design and setting:** a facility based cross-sectional study was conducted between March 2024 and March 2025 in the North-West Region of Cameroon. Data were collected in eight diagnostic and treatment centres (DTCs) that were purposively selected due to their high tuberculosis caseloads. These facilities accounted for more than 75% of all TB notifications in the region during the study period (Figure 1) [11]. Four of the selected facilities were public health institutions, while the remaining four were faith-based or confessional facilities.

**Study population:** the study population consisted of adult TB patients aged 18 years and above who were receiving treatment for either pulmonary or extrapulmonary tuberculosis in the selected DTCs during the study period. Only patients who were in the continuation phase of treatment and who provided informed consent were included. Patients who were severely ill or unable to participate in an interview were excluded from the study.

**Sample size determination:** the sample size was calculated using Cochran´s formula for cross-sectional studies [12]. Assuming a 95% confidence level (Z=1.96), an estimated proportion (p=0.5) due to the absence of prior regional data on tuberculosis knowledge and treatment adherence, and a margin of error of 5% (d=0.05), the initial sample size obtained for an infinite population was 384 participants. Because the total number of TB patients across the eight selected facilities was 705, a finite population correction was applied, resulting in an adjusted minimum sample size of 249 participants. To account for potential non-response, a 10% buffer was added, resulting in a target sample size of 294 participants.

Despite the ongoing socio-political instability in the region, including security concerns and transportation constraints, a total of 274 TB patients were successfully recruited, corresponding to a response rate of 93.2% of the target sample (Table 1). Participants were recruited consecutively as they attended routine follow-up visits at the selected diagnostic and treatment centres during the study period. The number of participants recruited from each facility was proportionally allocated according to the TB case load of each facility, ensuring that high-volume centres contributed proportionately to the overall sample.

**Table 1:** sample size calculation across eight selected diagnostic and treatment centers in North-West Cameroon, based on TB caseload and adjusted for finite population correction, with a 10% buffer added for non-response from March 2024 to March 2025

Facility	TB caseload 2024	Percent of total caseload	Minimum required after FPC	Target sample (10% buffer)	Actual recruitment	Percent of target
Bamenda Regional Hospital	200	28.4	71	78	73	93.6
Banso Baptist Hospital	55	7.8	19	21	20	95.2
Mbingo Baptist Hospital	104	14.8	37	41	38	92.7
Ndop District Hospital	54	7.7	19	21	20	95.2
Nkambe Regional Hospital Annex	108	15.3	38	42	39	92.9
Nkwen Baptist Health Centre	76	10.8	27	30	28	93.3
St. Elisabeth General Hospital Shisong	79	11.2	28	31	29	93.5
Wum District Hospital	29	4.1	10	11	10	90.9
Total	705	100	249	294	274	93.2

TB: tuberculosis; FPC: finite population correction

**Data collection instrument:** data was collected using a structured questionnaire developed for this study. The questionnaire was adapted from previously validated instruments used to assess tuberculosis knowledge and treatment adherence, including guidelines from the World Health Organization on adherence to long-term therapies [13]. The tool collected information on socio-demographic characteristics, tuberculosis knowledge, health-seeking behavior prior to diagnosis, treatment adherence, patient-reported experiences and barriers during treatment. The questionnaire was pretested among 20 TB patients at Tubah District Hospital, which was not included among the study sites, to assess clarity and consistency. Necessary adjustments were made before the main data collection.

### Operational definitions

**Tuberculosis knowledge:** knowledge was assessed using a series of questions covering TB transmission, symptoms, prevention, and treatment. Each correct response was assigned one point. The total knowledge score was calculated by summing the correct responses. Participants scoring at least 70% of the total score were categorized as knowledgeable.

**Treatment adherence:** treatment adherence was defined as self-reported intake of at least 90% of prescribed anti-tuberculosis medication doses during the previous 30 days. This information was cross-checked with clinic attendance records, where available. Participants reporting intake below this threshold were classified as non-adherent.

**Health-seeking behavior:** health-seeking behavior was defined as the actions taken by patients from the onset of TB-related symptoms to the time they sought care at a formal health facility. This included initial responses to symptoms, the type of provider first consulted, and delays before seeking medical care.

**Patient-reported experiences:** patient-reported experiences referred to challenges encountered during TB diagnosis and treatment, including medication side effects, transportation difficulties, and financial barriers.

**Data analysis:** data were entered and analyzed using Stata version 16. Descriptive statistics, including frequencies and percentages, were used to summarize socio-demographic characteristics, tuberculosis knowledge, health-seeking behaviour, and treatment adherence. Bivariate analysis using chi-square tests was conducted to examine associations between independent variables and treatment adherence. Variables with a p-value less than 0.20 in the bivariate analysis were included in the multivariable logistic regression model to identify independent predictors of treatment adherence. Adjusted odds ratios (AOR) with corresponding 95% confidence intervals were reported, and a p-value of less than 0.05 was considered statistically significant.

**Ethical considerations:** ethical approval was obtained from the Regional Ethics Committee for Human Health Research, under the Regional Delegation of Public Health for the North-West Region (Ref: 2024/05/12/CERSH-NW). Written informed consent was obtained from all participants. Data confidentiality and participants' anonymity were strictly maintained throughout the study. All eligible patients who were approached during the study period provided written informed consent, resulting in a 100% participation rate.

## Results

**Socio-demographic characteristics of study participants:** a total of 274 participants with tuberculosis were enrolled in the study. The median age was 41 years (interquartile range [IQR]: 30-44). Of the participants, 159 (58.0%) were male, and 115 (42.0%) were female (Table 2).

**Table 2:** sociodemographic characteristics of adult tuberculosis patients (N=274) enrolled in the study across eight high-volume diagnostic and treatment centers in North-West Cameroon from March 2024 to March 2025

Variable	Frequency
**Age (years)**	
15-24	38
25-34	61
35-44	55
45-54	48
55-64	43
65+	29
**Gender**	**Frequency (n, %)**
Male	159 (58.0%)
Female	115 (42.0%)
**Education**	**Frequency (n, %)**
No formal/primary	89 (32.7)
Secondary	149 (54.8)
University	34 (12.5)
**Marital status**	**Frequency (n, %)**
Unmarried	256 (93.4%)
Married	18 (6.6%)
**Employment**	**Frequency (n, %)**
Employed	103 (37.6%)
Unemployed	171 (62.4%)
**Treatment center**	**Frequency (n, %)**
Public DTC	164 (59.9%)
Faith-based	110 (40.1%)

The 'unmarried' category is a composite group that includes individuals who reported their status as single, widowed, or divorced; DTC: diagnostic and treatment centers

**Tuberculosis knowledge among patients:** male participants had significantly higher odds of being knowledgeable about tuberculosis compared to female participants (AOR = 3.57; 95% CI: 1.82-4.97; p<0.001). Educational level was also significantly associated with TB knowledge; participants with secondary education had higher odds of being knowledgeable than those with no formal or primary education (AOR = 1.50; 95% CI: 1.08-2.08; p=0.016). In contrast, occupation and marital status were not significantly associated with TB knowledge. Patients attending faith-based DTCs were significantly less likely to be knowledgeable about TB compared to those attending public facilities (AOR = 0.58; 95% CI: 0.34-0.98; p=0.042) (Table 3).

**Table 3:** tuberculosis knowledge among study participants (N=274) in North-West Cameroon, analyzed using binary and multivariate logistic regression from March 2024 to March 2025

Variable	Knowledgeable n (%)	Non-knowledgeable n (%)	OR	95% CI	P-value	AOR	95% CI	P-value
**Gender**								
Male (Ref.)	95 (59.7%)	64 (40.3%)	1.00	-	-	1.00	-	-
Female	38 (33.0%)	77 (67.0%)	0.33	0.20-0.55	<0.001	3.57	1.82-4.97	<0.001
**Education level**								
None/primary (Ref.)	23 (31.5%)	50 (68.5%)	1.00	-	-	1.00	-	-
Secondary	93 (69.9%)	40 (30.1%)	5.04	2.72-9.33	<0.001	1.50	1.08-2.08	0.016
University	17 (94.4%)	1 (5.6%)	38.9	4.9-308.9	<0.001	-	-	-
**Occupation**								
Unemployed (Ref.)	83 (48.5%)	88 (51.5%)	1.00	-	-	1.00	-	-
Employed	50 (48.5%)	53 (51.5%)	1.00	0.61-1.64	0.999	0.74	0.45-1.22	0.240
**Treatment center**								
Public DTC (Ref.)	93 (56.7%)	71 (43.3%)	1.00	-	-	1.00	-	-
Faith-based	40 (36.4%)	70 (63.6%)	0.44	0.26-0.73	0.001	0.58	0.34-0.98	0.042
**Marital status**								
Unmarried (Ref.)	126 (49.2%)	130 (50.8%)	1.00	-	-	1.00	-	-
Married	7 (38.9%)	11 (61.1%)	0.66	0.24-1.79	0.414	-	-	-

Knowledgeable: ≥ 70% correct answers

**Figure 2:**
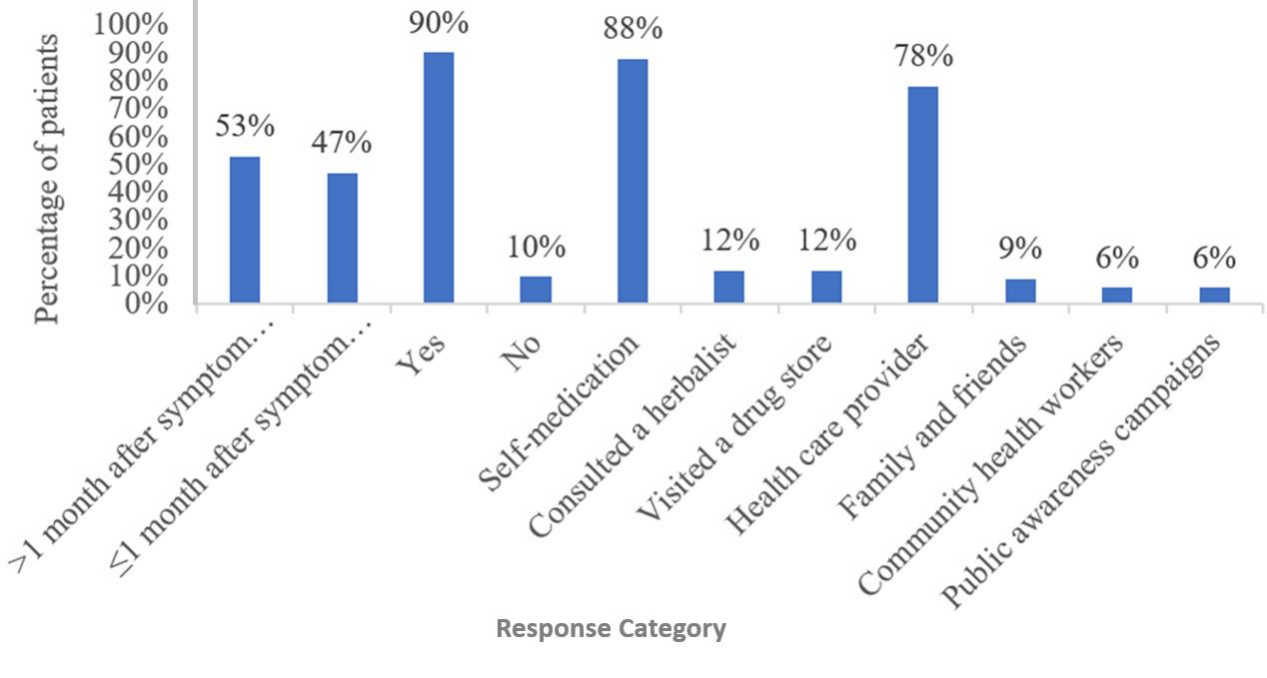
health-seeking behavior and TB awareness among patients in North-West Cameroon

**Health-seeking behavior:** health-seeking behavior related to TB differed across several components of TB control and prevention, including early symptom recognition, care-seeking practices, diagnostic testing, and treatment initiation. More than half of the participants were diagnosed more than one month after the onset of symptoms. Although most participants were eventually screened at a health facility, 88% reported practicing self-medication before seeking formal care, while a few consulted herbal practitioners or visited drugstores. When asked about their source of information on TB, more than two-thirds of participants cited a health care provider, while the rest either mentioned family and friends, community health workers, or reported public awareness campaigns as the primary source of information (Figure 2).

**Figure 3:**
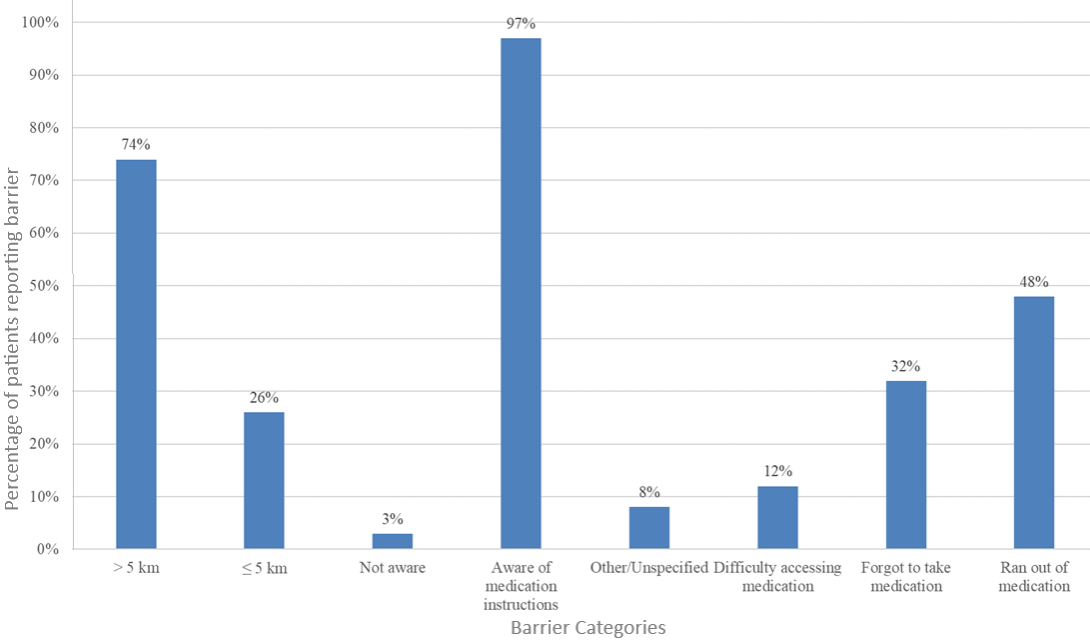
barriers to treatment adherence in North-West Cameroon

**Adherence to TB treatment:** regression analyses showed that gender was not a significant predictor of TB treatment adherence, although females had slightly higher odds of adherence compared with males. Educational level was significantly associated with adherence. Participants with secondary education were more likely to adhere to TB treatment compared with those with no formal or primary education, while university-level education showed no significant association. Employment status was also associated with adherence, with employed participants demonstrating higher odds of adherence compared with unemployed participants. Participants who ran out of medication were significantly less likely to adhere to TB treatment. The complete statistical results are presented in Table 4. To further explore factors affecting adherence, participants were asked about the practical challenges they faced during treatment. The most frequently reported barriers were running out of medication, forgetting doses, and difficulties accessing treatment centres. Although most participants were aware of the importance of maintaining regular TB medication doses, more than half lived more than five kilometres from a health facility (Figure 3).

**Table 4:** predictors of tuberculosis treatment adherence among study participants (N=274) in North-West Cameroon, analyzed using binary and multivariate logistic regression from March 2024 to March 2025

	Adherent n (%)	Non-adherent n (%)	OR	95% CI	P-value	AOR	95% CI	P-value
**Gender**								
Male (Ref.)	117 (73.6%)	42 (26.4%)	1.00	-	-	1.00	-	-
Female	75 (65.2%)	40 (34.8%)	0.67	0.40 - 1.12	0.126	0.77	0.46 - 1.28	0.320
**Education level**								
None/primary (Ref.)	47 (64.4%)	26 (35.6%)	1.00	-	-	1.00	-	-
Secondary	104 (78.3%)	29 (21.7%)	2.03	1.08 - 3.84	0.028*	1.87	1.24 - 2.81	0.003*
University	13 (72.2%)	5 (27.8%)	1.44	0.45 - 4.60	0.539	1.23	0.68 - 2.21	0.492
**Occupation**								
Unemployed (Ref.)	112 (65.5%)	59 (34.5%)	1.00	-	-	1.00	-	-
Employed	80 (77.7%)	23 (22.3%)	1.84	1.04 - 3.26	0.036*	1.06	0.63 - 1.78	0.839
**Marital status**								
Unmarried (Ref.)	180 (70.3%)	76 (29.7%)	1.00	-	-	1.00	-	-
Married	12 (66.7%)	6 (33.3%)	0.69	0.15 - 3.15	0.632	0.69	0.15 - 3.15	0.632
**Treatment center**								
Public DTC (Ref.)	119 (72.6%)	45 (27.4%)	1.00	-	-	1.00	-	-
Faith-based	73 (66.4%)	37 (33.6%)	1.50	0.87 - 2.60	0.147	1.50	0.87 - 2.60	0.147
**TB knowledge**								
Not knowledgeable (Ref.)	91 (64.1%)	51 (35.9%)	1.00	-	-	1.00	-	-
Knowledgeable	89 (66.9%)	44 (33.1%)	1.13	0.69 - 1.87	0.622	1.13	0.69 - 1.87	0.622

DTC: diagnostic and treatment center; *: variables that are statistically significant at p < 0.05

**Patient-reported experiences during TB treatment:** participants generally reported good communication with health care workers. Most indicated that they had received adequate counselling regarding the timely initiation of treatment, treatment duration, follow-up procedures, and possible drug side effects. However, some participants who reported missing doses attributed this to medication side effects, which negatively affected their motivation and adherence to treatment (Table 5).

**Table 5:** patient-reported experiences during TB treatment (N=274) regarding communication with healthcare providers, satisfaction, and barriers to care across eight diagnostics and treatment centers in North-West Cameroon from March 2024 to March 2025

Variables	Frequency (%)
**Communication with providers**	
Acknowledged	240 (87.6%)
Not acknowledged	34 (12.4%)
**Satisfaction with services**	
Satisfied	274 (100%)
**Recommendation of facility**	
Acknowledged	274 (100%)
**Hospital schedule**	
Satisfied	274 (100%)
**Missed doses (<1 day - ≥7 days)**	
Experienced	118 (43.1%)
Not experienced	156 (56.9%)
**Transportation barrier**	
Experienced	112 (40.9%)
Not experienced	162 (59.1%)

## Discussion

This study examined tuberculosis knowledge, health-seeking behavior, treatment adherence, and patient experiences among TB patients in the North-West Region of Cameroon. The findings provide insight into behavioral and structural factors influencing TB control in a conflict-affected setting where disruptions to health services can affect timely diagnosis and treatment continuity.

**Socio-demographic characteristics:** the majority of participants were male (58%), with a median age of 41 years. Most participants were aged 30-44 years, reflecting a pattern observed in studies conducted in Enugu, Nigeria, where tuberculosis disproportionately affects the economically active population [14]. A considerable proportion of participants had secondary education (53.8%), while nearly one-third had no formal or only primary education. In addition, most participants were unemployed (61.3%). These characteristics highlight the socioeconomic context in which TB occurs and may partly explain delays in care seeking, limited knowledge of TB, and challenges with treatment adherence [15,16].

**Knowledge of tuberculosis:** only 48.5% of participants were classified as knowledgeable about tuberculosis. Although awareness of TB symptoms was relatively high, misconceptions regarding disease transmission were common, including beliefs that tuberculosis could be transmitted through sexual contact or sharing utensils. Similar patterns of partial tuberculosis knowledge have been reported in a study conducted at Gimbi General Hospital, Ethiopia, where patients demonstrated awareness of symptoms but limited understanding of disease transmission and prevention [17].

Male participants and those with higher education levels were significantly more likely to be knowledgeable about TB. Regression analysis showed strong associations between male gender and TB knowledge as well as between education level and TB knowledge. Similar associations have been reported in studies conducted in Nigeria, Malawi, and Bangladesh, where education was a major determinant of TB knowledge [8,18,19]. Education likely improves access to health information and enhances understanding of disease prevention and treatment.

Interestingly, unmarried participants demonstrated slightly higher levels of TB knowledge compared with married participants. This difference may reflect variations in health information access or differences in autonomy related to health-seeking behavior. Similar observations have been reported in Bangladesh, where marital status was associated with health literacy [20].

**Health-seeking behavior:** this study identified significant delays in TB diagnosis. More than half of the participants were diagnosed more than one month after symptom onset. Diagnostic delays increase the risk of continued transmission and may worsen treatment outcomes, particularly in high-burden settings.

Self-medication before seeking formal care was common, with 88% of participants reporting the use of drugs from pharmacies or informal providers before visiting a health facility. Similar patterns have been reported in studies conducted in Peru and Ethiopia, where patients often delay formal care while attempting self-treatment [21,22]. Such behavior is frequently influenced by accessibility, financial constraints, perceived severity of symptoms, and cultural beliefs. Health care providers were the primary source of TB information for most participants (78%), followed by family members, community health workers, and public awareness campaigns. This finding highlights the central role of health workers in disseminating TB information and supporting patient education.

**Treatment adherence:** the treatment adherence rate in this study was 70.1%. Although this level of adherence is encouraging, it remains below the optimal levels required for effective TB control. The 2023 global TB report highlighted health system disruptions and medication shortages as key barriers to treatment continuity in many settings [2]. Education level and employment status were both significantly associated with treatment adherence. Participants with secondary education and those who were employed demonstrated higher adherence rates. Education may improve understanding of the importance of completing treatment, while employment may provide financial resources needed to cover transportation and other indirect costs associated with treatment.

Medication shortages were the most frequently reported barrier to adherence, followed by forgetfulness and difficulties accessing treatment centres. In addition, more than half of the participants lived more than five kilometers from a health facility, which may further contribute to challenges in maintaining regular treatment visits. Similar barriers have been documented in studies conducted in Ghana, where socioeconomic barriers and drug access influenced treatment completion [23]. Strengthening drug supply systems and decentralizing TB services could improve adherence in conflict-affected regions.

Interestingly, no significant association was observed between TB knowledge and treatment adherence. This suggests that knowledge alone may not be sufficient to ensure treatment compliance when structural barriers such as medication shortages and transportation challenges exist. Similar findings have been reported in Indonesia, where health system constraints played a larger role than patient knowledge in determining treatment outcomes [24]. Comparable findings have also been reported in Cameroon, where non-adherence to tuberculosis treatment was associated with socioeconomic and health system-related challenges.

**Patient-reported experiences during TB treatment:** most participants reported positive interactions with health care providers. A large majority confirmed receiving counselling on the importance of early treatment initiation and adherence to medication schedules. High levels of patient satisfaction were also reported, particularly regarding staff friendliness and accessibility of care. Despite these positive experiences, structural barriers remained evident. Medication shortages and long distances to treatment centres were frequently reported challenges. These barriers may compromise continuity of care and highlight the need for strategies that improve drug availability and expand decentralized TB services.

**Limitations:** this study has several limitations. First, the cross-sectional design limits the ability to establish causal relationships between tuberculosis knowledge, health-seeking behaviour, and treatment adherence. Second, treatment adherence was primarily assessed using self-reported information, which may be affected by recall bias or social desirability bias. Third, the study was conducted in selected high-volume diagnostic and treatment centres, which may limit the generalizability of the findings to all TB patients in the North-West Region. Finally, the ongoing socio-political instability in the region may have influenced patient access to care and participation during the study period.

**Priority actions to healthcare delivery should include:** establish satellite drug depots within 5 km of high-volume diagnostic and treatment centers to ensure sustained access to medication; gender-sensitive health promotion training programs should be introduced to address disparities in TB knowledge amongst the population; innovative strategies such as mobile health and adaptable adherence frameworks in conflict settings could be evaluated to improve TB control activities in vulnerable settings.

## Conclusion

This study, conducted in the North-West Region of Cameroon, identified important gaps in tuberculosis knowledge and suboptimal treatment adherence among TB patients in a conflict-affected setting. Only 48.5% of participants demonstrated adequate knowledge of TB, while treatment adherence was 70.1%, indicating remaining challenges for effective TB control. Educational level and employment status were associated with improved treatment adherence, suggesting that socioeconomic factors influence patients´ ability to complete treatment. However, structural barriers such as medication shortages, long distances to treatment centres, and transportation challenges appeared to have a stronger influence on adherence than TB knowledge alone. The ongoing socio-political crisis in the region continues to affect healthcare delivery and access to tuberculosis services. Addressing these challenges will require context-specific and patient-centred interventions aimed at improving treatment access and continuity of care. Strengthening drug supply systems, expanding decentralized treatment services within communities, and implementing targeted health education interventions may help improve treatment adherence and overall TB control in conflict-affected regions.

### What is known about this topic

- Tuberculosis remains a major public health problem globally, with significant challenges related to delayed diagnosis, treatment adherence, and health system barriers in many low and middle-income countries;
- Delays in seeking care and treatment interruptions remain common challenges in many high-burden TB settings;
- Structural barriers such as limited access to health facilities, medication availability, and socioeconomic constraints can negatively affect tuberculosis treatment outcomes in resource-limited settings.

### What this study adds

- This study provides empirical evidence on tuberculosis knowledge, health-seeking behaviour, and treatment adherence among TB patients in the conflict-affected North-West Region of Cameroon, a setting where limited data currently exist;
- The study identifies key predictors of tuberculosis knowledge and treatment adherence, particularly the role of educational level and employment status among TB patients;
- The findings highlight important structural barriers affecting treatment adherence, including medication shortages and long distances to treatment centres, emphasizing the need for context-specific interventions to improve tuberculosis care delivery in fragile health system settings.
